# Microbiological, Functional, and Chemico-Physical Characterization of Artisanal Kombucha: An Interesting Reservoir of Microbial Diversity

**DOI:** 10.3390/foods13121947

**Published:** 2024-06-20

**Authors:** Joel Armando Njieukam, Marianna Ciccone, Davide Gottardi, Arianna Ricci, Giuseppina Paola Parpinello, Lorenzo Siroli, Rosalba Lanciotti, Francesca Patrignani

**Affiliations:** 1 Department of Agricultural and Food Sciences, Campus of Food Science, Alma Mater Studiorum, University of Bologna, Piazza Goidanich 60, 47521 Cesena, Italy; joelarmando.njieuka2@unibo.it (J.A.N.); marianna.ciccone2@unibo.it (M.C.); davide.gottardi2@unibo.it (D.G.); arianna.ricci4@unibo.it (A.R.); giusi.parpinello@unibo.it (G.P.P.); rosalba.lanciotti@unibo.it (R.L.); francesca.patrignani@unibo.it (F.P.); 2 Interdepartmental Centre for Agri-Food Industrial Research, Campus of Food Science, Alma Mater Studiorum, University of Bologna, Via Quinto Bucci 336, 47521 Cesena, Italy

**Keywords:** kombucha, metagenomics, functionality, antioxidant activity, volatile molecule profile

## Abstract

Kombucha is a trending tea fermented via a complex microflora of yeasts and acetic acid bacteria. It can be a valid low-calorie substitute for soft drinks due to its sour, naturally carbonated, and sweet taste. Despite increased interest, the microflora and functional properties of kombucha have not yet been fully understood. The aim of this work was to characterize, from a microbiological, chemico-physical, and functional point of view, three types of artisanal kombucha obtained by fermenting green tea containing sugar by means of different starter cultures. Metagenomic analysis revealed a predominance of yeasts compared to bacteria, regardless of the sample. In particular, *Brettanomyces* spp. was found to be the dominant yeast. Moreover, the different types of kombucha had different microbial patterns in terms of acetic acid bacteria and yeasts. Ethanol and acetic acid were the dominant volatile molecules of the kombucha volatilome; the samples differed from each other in terms of their content of alcohols, esters, and acids. All the samples showed a high antioxidant potential linked to the high content of phenols. This study confirmed the positive chemico-physical and functional properties of kombucha and indicated that the microflora responsible for the fermentation process can significantly affect the characteristics of the final product.

## 1. Introduction

The consumer interest in functional fermented foods and drinks with beneficial health effects has significantly increased in recent years [1]. An example of a fermented beverage that is attracting considerable interest due to its functional properties is kombucha. In fact, it represents a valid substitute to soft drinks due to its acidic, naturally carbonated, and sweet taste, but with lower calories [2]. Kombucha is made starting from black or green tea infusions supplemented with sugars and a symbiotic culture of bacteria (specifically acetic acid and lactic acid bacteria) and yeasts (SCOBY) [3,4]. Kombucha is rich in bioactive compounds including natural antimicrobials, phenolic compounds (i.e., catechins and other flavonoids), amino acids (in particular, theanine), organic acids, and vitamins (E, K, B, and C), coupled with other micronutrients [3,5]. The potential health benefits of kombucha include antioxidant, antitumoral, antidiabetic, hepatoprotective, and antimicrobial properties [3]. However, most of the reported benefits should be proven via human clinical trials in order to determine whether kombucha consumption can be targeted to address specific health concerns [1].

The fermentation process lasts from 7 to 14 days at temperatures ranging between 20 and 30 °C [6,7]. Traditionally, kombucha is fermented in the presence of indigenous microorganisms, mainly yeasts (*Brettanomyces* spp., *Hanseniaspora* spp., *Saccharomyces* spp., *Pichia* spp., or *Zygosaccharomyces* spp.) and acetic acid bacteria (*Komagataeibacter* spp. *Acetobacter* spp., and *Gluconacetobacter* spp.), under aerobic conditions, and the resulting product is rich in organic acids (acetic acid, lactic acid, gluconic acid, and glucuronic acids) and CO_2_ [8]. Several parameters, such as the fermentation time and temperature, sugar content, type of tea, and use of starter cultures, as well as environmental factors (geographic place) affect the microbial composition of kombucha [9]. In this system, yeasts and bacteria cooperate and compete inside kombucha, and their metabolisms strongly affect the chemical composition of the final product [10]. Consequently, kombucha microbiota and their dynamics during the fermentation process affect both the sensory and functional characteristics of the product [11], and high microbial variability depends on production conditions [12,13]. In the production of kombucha, it is customary to introduce both the SCOBY and a portion of the fermented liquid from the previous batch into the sweetened tea [14]. However, it is possible to produce kombucha without the addition of a SCOBY but only with the fermented liquid from the previous batch.

Therefore, the aim of this work was to characterize, from a microbiological, functional, and chemico-physical point of view, three different batches of kombucha obtained via the inoculum of the fermented liquid from a previous batch in order to evaluate the effect of the starter used on the final quality of kombucha. Specifically, the samples were analyzed during and at the end of the production process from a microbiological point of view, using both culture-dependent and independent (metagenomic) methods. In addition, physico-chemical and functional characteristics, as well as the total phenol content, antioxidant activity, and volatile molecule profiles, were analyzed.

## 2. Materials and Methods

### 2.1. Kombucha Fermentation

The kombucha samples were provided by a local artisanal producer (Frui-Lab, Bologna, Italy). Three different kombucha batches, P1, P2, and P3, were produced through different blending and aromatization techniques to obtain commercial products with different characteristics. The starter inoculation was performed in the absence of a SCOBY, utilizing only the fermented liquid derived from the prior batch.

The starter of batch P1 was obtained from spontaneously fermented sweetened sencha green tea to which was added ginger and beetroot, which was then periodically backslopped by the producer.

The starters of batches P2 and P3 were originally two different commercial starters periodically backslopped by the producer.

The different batches had the same ingredients, sencha green tea and sucrose, 10%, and they were obtained by adding the three different microbial starters used by the manufacturer (P1, P2, and P3). The kombucha batches were prepared by adding 5 g of dried sencha green tea into 1 L of deionized hot water for 10 min. The resulting hot tea, to which was added 10% sucrose, was used as a fermentation medium for kombucha production (sugared raw tea).

In all cases, the inoculum was performed with the liquid part of a previous batch (50% of the total) of the same type of kombucha.

All the fermentations were carried out in 150 L plastic fermenters, with a fermentation volume of 100 L. Fermentation was conducted at a controlled temperature of 25 °C for 5 days. Samples were collected immediately after the inoculum (T_0_), at 48 h of fermentation (T_48_), and at the end of fermentation (T_end_).

### 2.2. Microbial Analysis Using Culture-Dependent Methods and Subsequent Identification of the Microbial Isolates

Ten mL of kombucha samples were diluted (range: 1:100–1:1,000,000) in a sterile saline solution (0.9% NaCl). The dilutions were plated on media selective for acetic acid bacteria (AAB), lactic acid bacteria (LAB), and yeasts. Specifically, for the detection of AAB, Gluconobacter agar medium-GA (25 g/L of mannitol, 5 g/L of yeast extract, 3 g/L of peptone, and 15 of g/L agar), glucose yeast extract agar—GYA (50 g/L of glucose, 5 g/L of yeast extract, and 15 g/L agar), acetic acid medium agar—AA (10 g/L of glucose, 5 g/L of ethanol, 3 g/L of acetic acid, 15 g/L of peptone, 8 g/L of yeast extract, and 15 g/L of agar) were used, all supplemented with cycloheximide as a selective agent. Yeast extract peptone dextrose (YPD) (Oxoid, Basingstoke, UK) with chloramphenicol added was used for the enumeration of yeasts, while LAB were enumerated in de Man Rogosa and Sharpe (MRS) medium (Oxoid, Basingstoke, UK) to which cycloheximide was added. Plates for the detection of AAB and yeasts were incubated at 25 °C for 24–48 h, while MRS agar plates were incubated at 30 °C for 24 h. Microbiological analyses were performed immediately after the inoculum (T_0_), at 48 h of fermentation (T_48_), and at the end of fermentation (T_end_).

The GA, GYA, AA, YPD, and MRS media allowed for the isolation of different colonies characterized by distinct morphologies. Afterwards, isolates were cultivated in their appropriate media and conditions for 24 h and then re-streaked onto appropriate agar media. Genomic DNA was extracted from each isolate using the InstaGene Matrix kit (Bio-Rad Laboratories, Milano, Italy). A total of 35 representative isolates were identified using RAPD-PCR (primer M13) and by sequencing the 16S and ITS1-ITS4 rRNA region for bacteria and yeasts, respectively, according to the protocol described by Siroli et al. [15].

### 2.3. Metagenomic Analyses

Metagenomic DNA from liquid kombucha samples was extracted at the beginning (T_0_) and end of fermentation (T_end_). gDNA was extracted with ZymoBIOMICS DNA Mini Kit (Zymo research, Irvine, CA, USA), and it was quantified with a spectrophotometer and a fluorimetric method using Qubit™ dsDNA HS Assay Kit (ThermoFisher Scientific, Waltham, MA, USA). Then, 20–200 ng/g of DNA was sheared in 10 mM of Tris-HCl with sonication to a fragment size ranging from 100 to 1000 bp. DNA libraries were generated following the standard protocol, including End Repair, dA-Tailing, Adaptor Ligation, and PCR Enrichment NEBNext^®^ Ultra™ II DNA Library Prep Kit for Illumina^®^ (New England Biolabs, Ipswich, MA, USA). The libraries were quantified with a Qubit™ dsDNA HS Assay Kit and analyzed with a High Sensitivity D1000 ScreenTape Assay kit (Agilent Technologies, Santa Clara, CA, USA) and the Agilent TapeStation 4200 instrument (Agilent Technologies, Santa Clara, CA, USA). The libraries were sequenced on an Illumina NovaSeq 6000 instrument (Illumina, San Diego, CA, USA) with paired-end reads 150 bp long. Fastq files were quality-tested using FastQC (https://www.bioinformatics.babraham.ac.uk/projects/fastqc/, accessed on 1 June 2024). A metagenomic analysis was performed using the CCMetagen pipeline, v. 1.3 [16].

### 2.4. Physico-Chemical and Functional Analyses

Samples were analyzed for ethanol (%), density (d), total and volatile acidity (g/L), organic acids (g/L, malic, lactic, and citric), sugars (g/L, glucose, and fructose), potassium (g/L), and catechins (g/L) using the multiparameter analyzer Bacchus 3 (Steroglass Srl, Perugia, Italy), consisting of a is5 FTIR Thermo Fisher Scientific spectrophotometer (Walthman, MS, USA) equipped with a hydraulic system for automatic sampling, a filter unit, and a “Bacchus Analysis” software (version 3) interface for analysis management. The pH (units) analysis was carried out using a pH meter (BASIC 20, Crison, Modena, Italy). The gluconic acid (mg/L) was determined with an enzymatic assay kit (K-GATE, Megazyme). Antioxidant activity (%) was evaluated through 2,2-diphenyl-1-picryl-hydrazyl (DPPH) by diluting samples in 80% methanol. For the calculation of the μmol of DPPH radical scavenged via the extracts, the absorbance value was measured at 517 nm after 10 min. A blank reagent was used to verify the stability of the DPPH radical dot during the test time. The kinetics of the antioxidant reaction were also determined over 30 min and compared with gallic acid as an antioxidant reference and expressed as mg/L of gallic acid equivalent.

The analysis of total phenolic compounds was carried out according to the Folin–Ciocalteu methodology, as reported by Slinkard and Singleton [17]. The mixture absorbance was read at 750 nm, and the concentration of total phenolic compounds was expressed as mg/L of gallic acid equivalents.

### 2.5. Volatile Molecule Profiles

Volatile molecule profile analyses were conducted on the kombucha samples using gaschromatography–mass spectrometry (GC-MS), coupled with a solid-phase microextraction (SPME) technique, according to Siroli et al. [18]. The samples (3 mL, placed in sterile vials), were added to 6 μL of standard 4-methyl-2-pentanol at 10,000 mg/kg and heated for 10 min at 45 °C. A Carboxen/polydimethylsiloxane (Carboxen/PDMS) fiber (85 μm, Stalleflex Supelco, Bellefonte, PA, USA) was exposed to the sample headspace for 30 min and then desorbed for 10 min in the injector of the GC, coupled with a quadrupolar mass-selective spectrometer (Agilent 7890 A GC and Agilent 5975 C MS, Agilent Technologies). Analytes were separated on a Chrompack CP-Wax 52 CB column (Chrompack, São Paulo, Brazil) using the conditions described by Siroli et al. [18]. The assignation of the volatile peaks was conducted by comparing the mass spectral data with those of the references present in the NIST library (NIST/EPA/NIH Mass spectral Library, Version 1.6, Gaithersburg, MD, USA) of 2011 and WILEY (sixth edition, New York, NY, USA) of 1995. Volatile compounds were quantified in mg/kg equivalents to the internal standard used (4-methyl-2-penthanol).

### 2.6. Statistical Analysis

The results are expressed as means of three independent measurements from three repeated experiments on different days. The data were statistically analyzed using a one-way ANOVA (statistica 8.0, Tulsa, OK, USA). The differences between mean values were detected via the HSD Tukey test, and evaluations were based on a significance level of *p* ≤ 0.05. Principal component analysis (PCA) was performed using the Statistica software, version 8.0, to obtain a visual overview of the volatile molecule profile of the different kombucha samples.

## 3. Results

### 3.1. Microbial Characterization of Kombucha Samples via Culture-Dependent Methods

**Figure 1:**
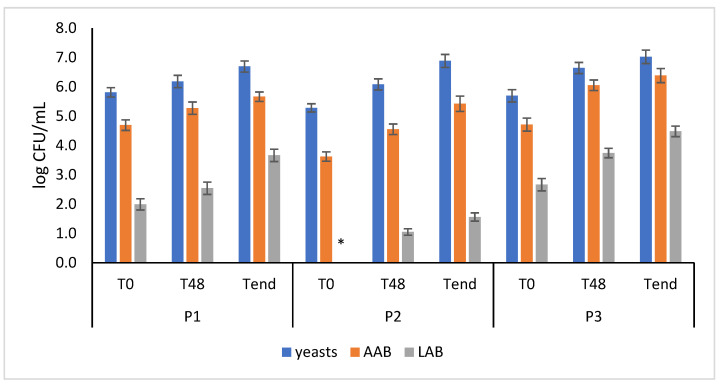
Cell load of yeasts, AAB, and LAB in the different kombucha samples (P1, P2, and P3) analyzed during the fermentation process (T_0_, T_48_, and T_end_). *: below the limit of quantification (<1 log CFU/mL).

The three different batches of kombucha (P1, P2, and P3) were analyzed during fermentation for microbiological patterns using culture-dependent methods. Figure 1 shows the cell load of AAB, LAB, and yeasts in the three types of kombucha after the inoculum (T_0_), 48 h later (T_48_), and at the end of the fermentation process, 120 h later (T_end_).

As shown in Figure 1, yeasts were found to be the dominant microflora independently of the type of kombucha and the time of sampling. They reached 6.7, 6.9, and 7.0 log CFU/mL in the P1, P2, and P3 samples, respectively, at the end of fermentation. The increase in the microbial load of the yeasts at the end of fermentation compared to the initial level ranged between 0.9 and 1.6 logarithmic cycles independently of the condition. Samples P1 and P3 showed an initial cell load of AAB (4.7 log CFU/mL) higher than P2 (3.6 log CFU/mL). All the samples increased the cell load of AAB during the fermentation process, with sample P3 reaching the highest cell load (6.4 log CFU/mL) of AAB at the end of fermentation. LABs were found on kombucha samples at lower levels than AAB and yeasts. Differences between the samples were found during and at the end of fermentation. The final LAB cell load was found to be 3.7, 1.6, and 4.5 log CFU/mL in samples P1, P2, and P3, respectively.

Colonies of different morphologies were isolated from the different culture media and identified. Among the 35 identified isolates, 15 were yeasts, 18 were acetic acid bacteria, and 2 were lactic acid bacteria (Table 1). The use of the traditional culture methods of isolation and identification resulted in the detection of limited biodiversity, especially in the case of yeasts that belonged only to the species *Starmerella davenportii* and *Brettanomyces bruxellensis*. *Starmerella* was detected in all samples and at all sampling times, while *Brettanomyces* was detected in all samples but only at the end of fermentation. Regarding LAB, only *Liquorilactobacillus nagelii* was detected in samples P1 and P3. AAB was detected in all samples, with *Acetobacter tropicalis* only at the beginning of fermentation and *Komagataeibacter* spp. at almost all sampling times, while *Gluconobacter oxydans* and *Komagataeibacter hansenii* were present only in samples P1 and P2, respectively.

### 3.2. Metagenomic Analyses

Also, culture-independent analyses (metagenomic) were performed on the kombucha samples (T_0_ and T_end_) since traditional culture methods for microbial population detection can have numerous limitations due to the presence of viable but not cultivable cells and fastidious microorganisms that can lead to an underestimation of real microbial populations. Table 2 shows, for each sample, the total number of readings and those attributable to the kingdom of bacteria and fungi, as well as their percentages of the total.

As can be seen from Table 2, the fungi kingdom was the dominant one in all the samples, with abundances at T_0_ of 98.83, 99.27, and 89.84% for P1, P2, and P3, respectively. Negligible reductions in fungi’s relative abundance were observed in the P1 and P2 samples at the end of fermentation, while sample P3 presented a drastic decrease in fungi’s relative abundance (final value: 69.9%). Bacteria were found to be less present than fungi, which was also in accordance with what was observed from traditional culture-dependent methods, and the ratio of fungi to bacteria was found to range between 84.2 and 31.5 for sample P1, between 135.6 and 150.7 for sample P2, and between 2.3 and 8.8 for sample P3.

**Figure 2:**
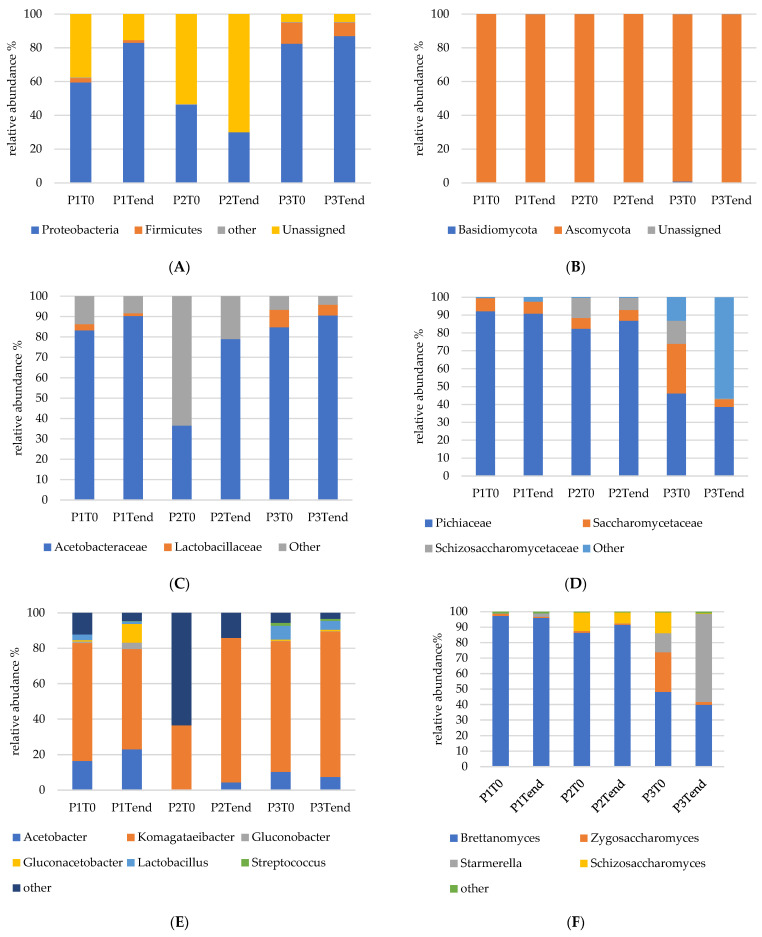

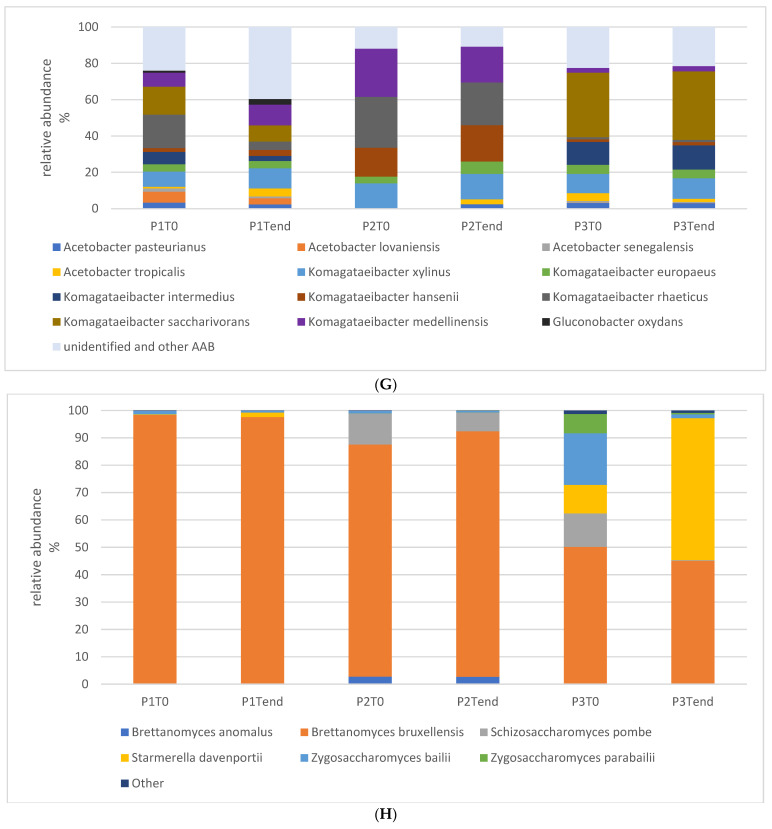
Relative abundance of bacterial (**A**) and fungal (**B**) phyla, bacterial (**C**) and fungal (**D**) families, bacterial (**E**) and fungal (**F**) genera, and bacterial (**G**) and fungal (**H**) species in different kombucha samples. Only phyla, families, and genera accounting for a minimum of 1% of reads in at least one sample were reported. For families and genera, unassigned bacteria and fungi were not considered.

Taxonomic assignments were performed at the phylum, family, genus and species levels. An average of 99.9% of fungi readings could be assigned at the phylum level, while, in the case of bacteria, the average percentage of assigned readings was found to be 73.5%, 38.3%, and 95.3%, respectively, for samples P1, P2, and P3. Figure 2 shows the relative abundance of fungi and bacteria phyla, families genera and species.

As shown in Figure 2A,B, Ascomycota accounted for over 99% of the fungal community in all samples considered, while Proteobacteria was the dominant bacterial phylum, accounting for 60% and 83% of sample P1, 46% and 30% of sample P2, 82% and 87% of sample P3 at the beginning and end of fermentation, respectively. A significant presence of Firmicutes was detected in samples P3 (12% and 8%) and P1 (3% and 2%), while sample P1 was characterized by the highest level of unassigned bacteria (53% and 70%).

Concerning fungi, at the family level (Figure 2D), Pichiaceae were the most abundant in samples P1 (90–91%) and P2 (82–86%), while in sample P3, they accounted only for 46% at T_0_ and 39% at the end of fermentation. Saccharomycetaceae were present at levels of about 6–7% in samples P1 and P2, regardless of the sampling time, whereas, in the P3 sample, they were present with an initial relative abundance of 28% and then decreased to 4% at the end of fermentation. Also, Schizosaccharomycetaceae were detected at percentages higher than 1%, but only in samples P2 and P3; in both cases, their relative abundance was higher at T_0_, at 11% and 12%, respectively, compared to the end of fermentation (6.8% and 0.4%, respectively). In sample P3, particularly at the end of fermentation, other fungi families also showed a high abundance (57%). At the genus level (Figure 2F), *Brettanomyces* spp. were found to be the predominant yeast in all the samples, especially in sample P1, in which its relative abundance was 97% at T_0_ and 95% at T_end_, and sample P2 (86% at T_0_ and 92% at T_end_). The *Brettanomyces* abundance was lower in sample P3, in which the initial abundance of 48% decreased to 40% at the end of fermentation. In sample P1, the only other representative genera whose relative abundances were more than 1% were *Zygosaccharomyces* spp., present at 1.5% at the initial time and 0.8% at the end of fermentation, and *Starmerella* spp., found at 2% at the end of fermentation. In sample P2, *Schizosaccharomyces* spp. were detected at a relative abundance of 12% and 7%, respectively, at T_0_ and T_end_, while *Starmerella* was present at 0.02% and 0.07%, respectively, at T_0_ and T_end_. In sample P3, *Starmerella* was found to be the most representative yeast at T_end_, showing a relative abundance of 57%. At the species level (Figure 2H), *B. bruxellensis* was predominant, accounting for over 97% of the P1 sample and over 85% of the P3 sample at both the beginning and end of fermentation. In the P2 samples, only *S. pombe* and *B. anomalus* were present at percentages higher than 2% at T_end_. In the case of the P3 samples, *B. bruxellensis*, *S. pombe*, *S. davenportii*, and *Z. bailii* were detected at the beginning of fermentation at percentages higher than 10%. However, by the end of fermentation, *S. davenportii* and *B. bruxellensis* accounted for 97% of the yeast population.

Regarding bacteria (Figure 2C,E), Acetobacteraceae were the most abundant family, and they increased over time up to levels of 90%, 79%, and 91% in P1, P2, and P3, respectively. Lactobacillaceae were found only in sample P1 (1–2%) and to a major extent in P3 (5–8%). At the genus level, *Komagataeibacter* spp. was the most representative genus in all the kombucha samples since its relative abundance at T_end_ was higher than 80% in samples P2 and P3, and it was 56% in sample P1. The relative abundance of *Acetobacter* spp. increased in samples P1 and P2 during fermentation and was detected at T_end_ at an abundance of 23%, 4%, and 7% in samples P1, P2, and P3, respectively. *Gluconobacter* spp. and *Gluconacetobacter* spp. were present at a relative abundance higher than 1% only in sample P1. Finally, *Lactobacillus* spp. was detected only in samples P1 and P3, while *Streptococcus* spp. was detected only in the P3 sample. At the species level (Figure 2G), in the P1 sample, *K. saccharivorans*, *K. rhaeticus*, *K. xylinus*, and *K. medellinensis* were the predominant species, but none of them were present at a percentage higher than 11%. However, these samples also showed high percentages of unidentified AAB. In the P2 samples, *K. rhaeticus* and *K. medellinensis* were the predominant AAB at the beginning of fermentation, while at the end of fermentation, in addition to these species, *K. hansenii* and *K. xylinus* were also present at percentages close to 20%. Concerning the P3 samples, *K. saccharivorans* was the dominant AAB at both T_0_ and T_end_ (>30%), but *K. xylinus* and *K. intermedius* also increased their presence at T_end_.

### 3.3. Physico-Chemical and Functional Analyses

Table 3 shows the chemical composition of the different kombucha samples, including the unfermented sencha green tea (control). An increase in alcohol content compared to the sugared raw material was observed in all the samples (P1, P2, and P3). The alcohol content ranged between 0.90% and 1.54%, regardless of the time of fermentation. P1 and P2 showed a higher alcohol content compared to P3, while no significant differences were observed within each batch.

At the end of fermentation, sample P3 was characterized by the lowest pH (2.76) and the highest total acidity (8.4 g/L). Samples P1 and P3 showed significantly higher volatile acidity (4.8 and 5.2 g/L, respectively) compared to sample P2 (3.4 g/L). Sample P3 at T_end_ showed a significant increase in malic acid, while in the other samples, the malic acid content did not deviate much from the T_0_ values. The lactic acid content did not change significantly in sample P1, while it increased in P2 and decreased in P3 at T_end_. Gluconic acid was not detected in the sencha green tea, while it increased at the end of fermentation in samples P1 and P3.

The glucose concentration was found to be higher in the P3 sample at both the beginning and end of fermentation (29–31 g/L), while it ranged between 21 and 24 g/L in samples P1 and P2. Fructose was found to be present in lower quantities than glucose (12–17 g/L), but in this case, a significant decrease was observed at T_end_ in samples P2 and P3. The glucose and fructose content was lower in the sugared sencha green tea (sugared raw tea). The highest concentration of sucrose was observed in the sugared raw tea. In the samples at T_0_, the concentrations ranged from 67 to 69 g/L. A reduction in the concentration of sucrose was observed in all samples at both T_48_ and T_end_. The residual sucrose at the end of fermentation was found to be higher in the P1 samples (20.6 g/L), followed by P2 (17 g/L) and P3 (14 g/L).

The potassium content was affected by the kombucha batch (P3 > P2 > P1). In particular, P3 samples at T_end_ showed a significant increase in potassium compared to the other samples. The catechin content did not change significantly during the fermentation process within each sample; however, samples P1 and P3 at the end of fermentation showed significantly higher catechin content compared to the sencha green tea and sample P2 at T_end_. Regarding the total phenolic compounds and DPPH scavenging activity, samples P1 and P3 showed a significant decrease in these parameters at the end of fermentation compared to the starting sencha green tea.

### 3.4. Volatile Molecule Profiles

Specific volatile molecule profiles were detected based on the considered kombucha sample (Table 4). A total of 37, 32, 31, and 21 volatile molecules were identified in the aromatic profiles of P1, P2, P3, and the sugared raw tea samples, respectively. The molecules detected in the kombucha samples belonged to the chemical classes of alcohols, acids, ketones, aldehydes, and esters. The initial concentration of volatile molecules in the sencha green tea was lower (6 ppm) than in the fermented samples (83–133 ppm).

In the kombucha samples, the predominant molecules were alcohols and acids, followed to a lesser extent by esters. In general, samples P2 and P3 showed a higher concentration of volatile molecules than P1. The total amount of volatile molecules did not increase over time in sample P1, while an increase in the amount of alcohols and acids was observed during fermentation in samples P2 and P3. The total amounts of alcohols, esters, and acids were already high at the beginning of fermentation. This is not surprising since the initial inoculum was performed with a 50% liquid phase of kombucha coming from a previous batch. In the kombucha samples, the characterizing molecules were acetic acid, ethanol, phenylethyl alcohol, ethyl acetate, and caprylic acid.

**Figure 3:**
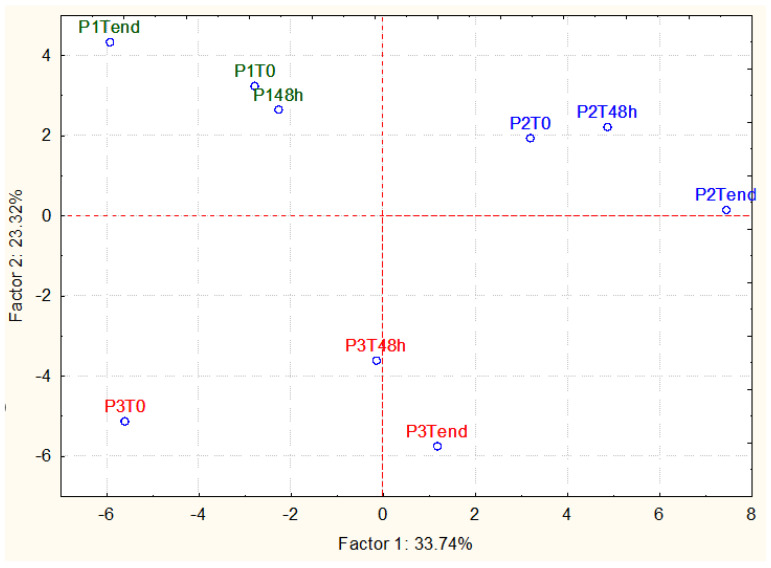
Projection of the different kombucha samples (P1, P2, and P3) on the factor plane (1−2) at different times of fermentation (T_0_, T_48_, and T_end_) on the basis of the volatile profiles detected via solid-phase microextraction combined with a chromatography-mass spectrometry technique (GC/MS SPME).

The GC-MS-SPME data were subjected to a principal component analysis (PCA) to better disclose the different kombucha samples. Figure 3 shows the projection of cases on the factorial plane (1 × 2) spanned by the first two factors (PC1 and PC2), where the main effect was the kombucha starter (P1, P2, and P3). The P1 samples were well separated from the P2 ones, mainly along PC1, which explains 33.74% of the variance, while they were well separated from those of P3, mainly along PC2, which explains 23.32% of the variance. The P2 samples, on the other hand, were well separated from the P3 samples both along the PC1 and, above all, along the PC2. In the case of the P3 sample, T_0_ was separated along PC1 from samples T_48_ and T_end_, which were found to be very similar. In contrast, samples P1 and P2 at T_0_ and T_48_ grouped together and were separated from sample T_end_ along both PC1 and PC2.

Regarding the molecules responsible for the clustering of the samples, sample P2 was characterized by a higher amount of esters (mainly ethyl acetate and ethyl octanoate) and alcohols (ethanol, isoamyl alcohol, 4-ethylguaiacol, and 4-ethylphenol). Sample P3 was characterized by the highest amount of acids (acetic acid) and the lowest amount of esters. Finally, the P1 sample showed the highest amount of short-chain fatty acids (caprylic acid, hexanoic acid, and decanoic acid).

## 4. Discussion

In this work, different samples of kombucha, obtained from an artisanal manufacturer (P1, P2, and P3) and at different steps/stages of fermentation (T_0_, T_48_, and T_end_) were characterized from a microbiological and chemico-physical point of view in order to understand the microbial biodiversity and differences in the samples’ compositions. Microbiological analyses were carried out using both traditional methods and a culture-independent metagenomic approach. Previous studies indicate that the microbial community in terms of yeasts and AAB abundance may vary between different kombucha fermentations across the globe, depending on the source and method of the inoculum used and the type of tea [19,20]. However, it is widely reported that AAB usually dominates the bacterial community of both SCOBY and the liquid phase of kombucha, driving the fermentative process [21]. Nevertheless, the microbiological data of the present work, deriving from culture-dependent and independent methods, have shown that yeasts were the predominant microflora in the liquid phase of the analyzed kombucha samples. On the other hand, the artisanal kombucha samples studied in this work were obtained from previous batches that were backslopped utilizing only the fermented liquid and not the SCOBY; this probably affected the microbial community, favoring a higher presence of yeasts than AAB. In fact, as reported by other authors, the larger amount of AAB would be entrapped in the pellicle on the surface of the kombucha [22,23]. Interestingly, the cell load of cultivable AAB differed based on the type of kombucha and ranged between 5.4 and 5.7 log CFU/mL in samples P1 and P2, while it increased considerably in sample P3 (6.5 log CFU/mL). The literature’s data indicate AAB levels ranging between 6.5 and 7.5 log CFU/mL in kombucha liquor [21]. Lactic acid bacteria were only detected in samples P1 and P3, and this was also confirmed via molecular methods. On the other hand, it is reported that lactic acid bacteria may be present in kombucha even though they do not represent the dominant microflora [24]. The microbial colonies included limited genera and species of yeasts, mostly identified as *Starmerella davenportii* and *Brettanomyces bruxellensis*. Among bacteria, *Acetobacter tropicalis*, *Komagataeibacter saccharivorans*, *Gluconobacter oxydans*, *Komagataeibacter hansenii*, and *Liquorilactobacillus nagelii* were identified. Certainly, some differences in the microorganisms identified via culture-dependent methods and metagenomic analysis were observed. As expected, metagenomic analysis showed a higher biodiversity within both the fungi and bacteria kingdoms. This is not surprising since the traditional culture-dependent methods used for microbial population enumeration have several drawbacks. For instance, the presence of viable but not cultivable cells and fastidious microorganisms may lead to an underestimation of the microbial population. On the other hand, metagenomic analyses allow for the observation of greater diversity in the microbial population [24]. In addition, culture-dependent methods can promote the growth of certain microbial species over others, as observed in this study. In fact, the metagenomic approach showed that *Brettanomyces* spp. was the dominant genus in kombucha samples P1 and P2, while *Starmerella* spp. and *Brettanomyces* spp. dominated the yeast microflora of sample P3. However, the samples also contained *Schizosaccharomyces* spp. and *Zygosaccharomyces* spp., with relative abundances that depended on the sample considered. The data obtained are in agreement with the literature; in fact, several species of *Saccharomyces*, *Saccharomycodes*, *Schizosaccharomyces*, *Zygosaccharomyces*, *Brettanomyces*, *Candida*, *Torulaspora*, *Kloeckera*, *Pichia*, *Mycotorula*, and *Mycoderma* have been associated with kombucha [25,26]. On the other hand, the yeasts population of kombucha can be extremely variable, depending on the production area of the starter used and the fermentation conditions [5,25]. At the species level, *Brettanomyces bruxellensis* was the predominant yeast in samples P1 and P2, while *Starmerella davenportii* was predominant in sample P3. *Brettanomyces bruxellensis* is widely reported among the predominant yeast species in the kombucha population [13,27] due to its high resistance to osmotic and ethanol stress [13]. There is less literature available on the impact of *Starmerella* on kombucha fermentation. This is partly because *Starmerella* is a relatively new genus, resulting from the reclassification of several *Candida* species. Consequently, earlier kombucha studies that identified *Candida* at the genus level might have included species now classified as *Starmerella* [22]. A *Starmerella davenportii* strain was recently isolated from kombucha, and it has been shown to produce volatile aroma compounds and organic acids during black tea fermentation, showing its capability to be a potential starter for kombucha production [28].

Metagenomic analyses revealed *Komagataeibacter* spp. as the dominant genus regardless of the kombucha sample and the time of fermentation considered, as previously reported in other studies [19,22]. In fact, kombucha represents the most important reservoir of the genus *Komagataeibacter*, widely described as cellulose-producing bacteria [29,30]. Interest in bacterial cellulose has increased considerably in recent years due to excellent technological properties, including high purity, water retention capacity, high mechanical strength, and biocompatibility [30]. In fact, the isolation and selection of the highly bacterial cellulose producers *Komagataeibacter* spp. have become a matter of primary attention [30]. However, *Acetobacter* spp., *Gluconacetobacter* spp., and *Gluconobacter* spp. were also detected. In the kombucha samples analyzed, specific species of AAB demonstrated distinct patterns of predominance. For the P1 sample, *K. saccharivorans*, *K. rhaeticus*, *K. xylinus*, and *K. medellinensis* were the predominant species. In the P2 sample, the AAB population was dominated by *K. rhaeticus*, *K. medellinensis*, *K. hansenii*, and *K. xylinus*. Conversely, the P3 sample was primarily composed of *K. saccharivorans*, *K. xylinus*, and *K. intermedius*. These findings highlight the variability in AAB communities across different kombucha samples, potentially influencing the fermentation process and the final products’ properties [13,19,31]. LAB were detected only in samples P1 and P3 with an abundance lower than 8%. According to the literature, their abundance in artisanal kombucha is negligible [5,19,21,31,32], while LAB can reach higher quantities in commercial products [33].

Sample P3 showed a higher load of AAB and the major biodiversity of microbial populations during fermentation. This resulted in a lower pH and higher volatile and total acidity for the P3 sample. However, all samples had pH values in line with the literature, generally between 2.5 and 3.8 [26,34]. A significant decrease in pH during fermentation was observed only in sample P3. This behavior can be attributed to the very high inoculum level (50% from a previous batch) used by the artisanal manufacturer, which lowered the initial pH of the kombucha. In all samples, a significant decrease in sucrose content over time was observed. Typically, sucrose is hydrolyzed by yeasts into fructose and glucose, which are then metabolized by yeast to produce ethanol and carbon dioxide. Additionally, due to the high osmotic pressure, glycerol is produced by yeasts [35]. Acetic acid bacteria can utilize glucose and fructose to primarily produce acids, including acetic acid, gluconic acid, and glucuronic acid. However, both glucose and fructose serve as substrates for the synthesis of the cellulose film by *Komagataeibacter* spp., which comprises the SCOBY [4,36]. Sample P3 showed an increase in the total and volatile acidity of fermented kombucha over time, while slight modifications were observed during fermentation in samples P1 and P2. The acidic content, particularly the content of volatile acids, such as acetic acid, is generally linked to fermentation conditions, particularly temperature and sugar concentrations, rather than to the type of microflora and, in particular, AAB [37]. At T_end_, sample P3 showed a significantly higher content of malic acid compared to the other samples. The same trend was also observed by Nie et al. [38] in the production of traditional vinegar. Other authors showed an increase in malic acid during kombucha fermentation associated with the activity of AAB [23,39]. Lactic acid was found at low concentrations in all samples and did not change throughout the fermentation process; this is consistent with the literature’s data reporting a very small increase in the concentration of lactic acid in kombucha fermentation using sucrose [40]. Conversely, the concentration of gluconic acid was dependent on the sample, with P3, which contained a higher amount of AAB, showing the highest level at the end of fermentation (T_end_). On the other hand, gluconic acid production is correlated with the activity of AAB, and its content depends on strain interactions, inoculum ratios, and inoculum sizes [21,41]. Gluconic acid not only enhances the health benefit of kombucha but also positively impacts the sensory and flavor properties of kombucha [41]. The amounts of glucose and fructose were dependent on the sample but did not undergo any major changes during fermentation. On the other hand, during kombucha fermentation, sucrose is converted into glucose and fructose and, further, into ethanol, acetic acid, lactic acid, and a large number of other compounds [40]. As reported by other authors, the sugar content of commercial kombucha per 100 mL ranged from 0.2 to 6 g [42]. The total sugar content observed in the analyzed samples resulted in this range. To qualify as low-calorie, kombucha should ideally have no more than 5 g of sugar per 240 milliliters [43]. Even though the sugar content in kombucha may overcome the limit to classify the beverage as a low-calorie soft drink, it is important to consider that kombucha is often unpasteurized and unfiltered. This allows microbial activity to continue during storage, leading to further sugar consumption. Additionally, kombucha can be subjected to flavor infusion or dilution processes, which can reduce its sugar content and, consequently, its caloric content. All the samples exceeded 1% alcohol by volume (ABV) at T_end_; this is in line with the literature’s data since naturally fermented kombucha may contain 0 to 3% ABV, depending on the microflora and fermentation conditions [44]. The European Union generally defines non-alcoholic beverages as those containing less than 0.5% ABV; however, each country has specific regulations. For example, in Italy and France, non-alcoholic beverages must contain less than 1.2% ABV [45]. However, kombucha may not be marketed as-is but is subjected to flavor infusion and dilution processes that allow it to meet the standards for non-alcoholic beverages.

All the samples showed a high content of total phenolic compounds and catechins associated with high antioxidant activity. The beneficial impact of phenolic compounds on human health is widely reported since they improve lipid profiles, blood pressure, insulin resistance, and systemic inflammation [46]. However, although differences were observed based on the sample type and fermentation time, all samples showed an increase in catechin content and a decrease in total phenolic compounds compared to the sugared raw tea. On the other hand, during the initial stages of kombucha fermentation, the concentration of catechins may decrease due to enzymatic oxidation and microbial metabolism. However, as fermentation progresses, the concentration of some catechins, such as epigallocatechin gallate, may increase due to the metabolic activities of AAB and yeast [36]. In addition, the concentration of total phenolic compounds was higher in the P2 samples, while during fermentation, it remained constant in all samples at T3 before showing a decrease at the end of the fermentation process. Antioxidant activity, on the other hand, was higher in the P1 and P2 samples. The literature data indicate that the antioxidant activity and total phenolic compound content of kombucha is strongly linked to the type of tea used in preparation (green tea has a greater antioxidant power than black tea) and to the duration of fermentation [47,48]. In general, an initial increase in antioxidant activity is observed, but during excessively prolonged fermentations, a decrease in this parameter can be detected [7,32]. The kombucha microbial consortium can cause chemical and structural alterations in phenolic acids due to biotransformation and metabolic activities [36].

The three types of kombucha analyzed showed specific profiles in volatile molecules. The aromatic compounds in kombucha can be affected by the tea constituents and derived ingredients, the sugar substrates, the microbial population, and the phase and temperature of fermentation [49]. This study showed a prevalence of acids, followed by alcohols, in the P3 sample, which had the highest abundance of AAB. This is in accordance with the literature’s data showing that carboxylic acids are also the major components of the fermentation process, followed by alcohols [49,50]. Acetic acid is normally produced by AAB via the oxidation of ethanol, and it contributes to the sour and distinctive flavor of kombucha [35]. Caprylic and hexanoic acid were found to be the most abundant carboxylic acids, and they were present in higher concentrations in samples P1 and P2, which were characterized by the highest presence of *Brettanomyces* spp. Other authors reported that fermentations with *Brettanomyces bruxellensis* produced the highest overall concentration of fatty acids, and their presence is related to yeast autolysis, which could be involved in the final yeasty aroma of kombucha, together with high alcohol [9,51]. Also, the presence of 4-ethylguaiacol, 4-ethylphenol was observed only in samples P1 and P2, and these molecules are generally produced by *Brettanomyces* spp., starting from coumaric acid [51]. High concentrations of these compounds are generally associated with the odor perception of horse sweat, barnyards, and medicine, and they are negatively perceived in wine and bottom-fermented pilsner beers [52,53]. However, their presence is essential for the overall flavor perception of many top-fermented blond and dark beers, as well as kefir, kombucha, and cider [54]. Together with ethanol, isoamyl alcohol and phenylethyl alcohol were the most abundant volatile alcohols, and their presence in kombucha is widely reported; they are produced from the respective amino acids via yeast activity [9]. Ethyl acetate was the most representative ester detected in accordance with the literature’s data [49]. The high presence of esters in fermented beverages is very important since it is generally associated with pleasant floral and fruity aromas, enhancing the tea and white fruit odor perception in kombucha [55].

## 5. Conclusions

Microbiological analyses revealed yeasts as the predominant microflora of the analyzed artisanal kombucha samples, contrary to the common belief that AAB dominate the kombucha microflora. This deviation could be attributed to the artisanal production method, through which only the fermented liquid, not the SCOBY, was utilized. The presence of yeasts such as *Starmerella davenportii* and *Brettanomyces bruxellensis* was notable, alongside various bacterial genera including *Acetobacter*, *Komagataeibacter*, and *Gluconobacter*. Chemico-physical analyses revealed variations in acidity, organic acids, and volatile compounds among the samples, influenced by microbial composition and fermentation conditions. Notably, a higher AAB load in sample P3 was correlated with increased total acidity, as well as malic and gluconic acid content. All the samples exhibited high polyphenol and catechin content with substantial antioxidant activity. Acids predominated the volatile profile of the samples, followed by alcohols and esters, contributing to the characteristic aroma and flavor of kombucha. PCA analysis showed that the three analyzed kombucha samples had specific and distinct profiles in volatile molecules and appeared well separated from each other in the factorial plan. Based on the results obtained, the P3 starter appears to be the most interesting one due to the more balanced microflora in terms of yeasts and lactic acid bacteria, resulting in a kombucha with a higher content of gluconic acid and a lower ethanol content, as well as a more balanced aromatic profile in terms of acids and alcohols. Overall, this study provides valuable insights into the microbial dynamics and chemical composition of artisanal kombucha, emphasizing the role of fermentation parameters and microbial interactions in shaping its properties. The present work also allowed the isolation of strains, such as those belonging to the genera *Komagataeibacter*, gifted with very interesting technological features that are potentially exploitable in the production of bio-based materials.

## Figures and Tables

**Table 1:** Isolated and identified strains from different kombucha samples at different sampling times.

Sample	Sampling Time	Strain Code	Identification
P1	T_0_	1MB	*Acetobacter tropicalis*
		1DA	*Komagataeibacter* spp.
		1YB	*Starmerella davenportii*
		2MB	*Acetobacter tropicalis*
		1YC	*Starmerella davenportii*
	T_48_	3DB	*Acetobacter tropicalis*
		3DA	*Gluconobacter oxydans*
		3YC	*Starmerella davenportii*
	T_end_	4MA	*Liquorilactobacillus nagelii*
		4MF	*Komagataeibacter* spp.
		4YF	*Brettanomyces bruxellensis*
		4YB	*Starmerella davenportii*
P2	T_0_	5YB	*Starmerella davenportii*
		6MA	*Acetobacter tropicalis*
		6YC	*Starmerella davenportii*
	T_48_	7DB	*Komagataeibacter* spp.
		7DC	*Komagataeibacter* spp.
		7MA	*Komagataeibacter hansenii*
		7YB	*Starmerella davenportii*
	T_end_	8DB	*Komagataeibacter* spp.
		8YE	*Brettanomyces bruxellensis*
		8YC	*Starmerella davenportii*
P3	T_0_	9DA	*Acetobacter tropicalis*
		9DB	*Acetobacter tropicalis*
		9YB	*Starmerella davenportii*
		10DA	*Komagataeibacter* spp.
		10MA	*Liquorilactobacillus nagelii*
		10YB	*Starmerella davenportii*
	T_48_	11DA	*Komagataeibacter* spp.
		11DB	*Komagataeibacter* spp.
		11YB	*Starmerella davenportii*
	T_end_	12YB	*Starmerella davenportii*
		12YF	*Brettanomyces bruxellensis*
		12DA	*Komagataeibacter* spp.
		12DB	*Komagataeibacter* spp.

**Table 2:** Relative abundance of bacteria, fungi, and bacteria/fungi ratio in Kombucha samples according to metagenomic analyses.

Sample	Sampling Day	Fungi (%)	Bacteria (%)	Fungi/Bacteria Ratio
P1	T_0_	98.83	1.17	84.5
	T_end_	96.93	3.07	31.6
P2	T_0_	99.27	0.73	135.9
	T_end_	99.34	0.66	150.5
P3	T_0_	89.84	10.16	8.8
	T_end_	69.94	30.06	2.3

**Table 3:** Chemical profiles of the different kombucha samples (P1, P2, and P3) at different times of fermentation (T_0_, T_48_, and T_end_) and sugared raw sencha tea.

	P1			P2			P3			
	T_0_	T_48_	T_end_	T_0_	T_48_	T_end_	T_0_	T_48_	T_end_	Sugared Raw Tea
Alcohol (%)	1.28 ± 0.14 ^cd^	1.32 ± 0.15 ^cd^	1.46 ± 0.16 ^d^	1.42 ± 0.16 ^d^	1.54 ± 0.17 ^d^	1.52 ± 0.17 ^d^	0.90 ± 0.10 ^b^	1.00 ± 0.11 ^b^	1.08 ± 0.12 ^bc^	0.45 ± 0.05 ^a^
Density (d)	2.0 ± 0.1 ^a^	2.0 ± 0.1 ^a^	2.0 ± 0.2 ^a^	2.0 ± 0.2 ^a^	2.0 ± 0.1 ^a^	2.0 ± 0.1 ^a^	2.0 ± 0.1 ^a^	2.0 ± 0.1 ^a^	2.0 ± 0.1 ^a^	2.0 ± 0.1 ^a^
Total acidity (g/L)	4.7 ± 0.3 ^c^	4.9 ± 0.3 ^cd^	5.0 ± 0.3 ^cd^	4.3 ± 0.5 ^bc^	3.7 ± 0.5 ^b^	4.5 ± 0.5 ^bc^	5.3 ± 0.4 ^cd^	5.5 ± 0.3 ^d^	8.4 ± 0.6 ^e^	0.30 ± 0.05 ^a^
pH (U)	3.07 ± 0.10 ^cd^	3.06 ± 0.08 ^cd^	3.06 ± 0.09 ^cd^	3.16 ± 0.10 ^d^	3.02 ± 0.11 ^bcd^	3.17 ± 0.08 ^d^	2.93 ± 0.07 ^bc^	2.88 ± 0.05 ^b^	2.76 ± 0.05 ^a^	7.46 ± 0.09 ^e^
Volatile acidity (g/L)	5.1 ± 0.3 ^d^	5.2 ± 0.2 ^d^	4.8 ± 0.3 ^d^	3.9 ± 0.2 ^c^	3.0 ± 0.3 ^b^	3.4 ± 0.3 ^bc^	3.5 ± 0.3 ^bc^	3.6 ± 0.3 ^bc^	5.2 ± 0.3 ^d^	0.11 ± 0013 ^a^
Malic acid (g/L)	2.22 ± 0.12 ^b^	2.24 ± 0.21 ^b^	2.18 ± 0.18 ^b^	2.08 ± 0.15 ^b^	1.72 ± 0.12 ^a^	2.28 ± 0.16 ^b^	2.82 ± 0.22 ^c^	2.92 ± 0.23 ^c^	4.50 ± 0.33 ^d^	*
Lactic acid (g/L)	0.44 ± 0.09 ^abc^	0.26 ± 0.09 ^a^	0.38 ± 0.08 ^ab^	0.44 ± 0.10 ^abc^	0.64 ± 0.08 ^d^	0.62 ± 0.07 ^d^	0.62 ± 0.10 ^cd^	0.52 ± 0.08 ^bcd^	0.26 ± 0.09 ^a^	0.56 ± 0.10 ^bcd^
Gluconic acid (g/L)	1.05 ± 0.12 ^b^	1.20 ± 0.08 ^bc^	1.27 ± 0.07 ^c^	0.30 ± 0.05 ^a^	0.31 ± 0.04 ^a^	0.42 ± 0.10 ^a^	1.21 ± 0.15 ^bc^	1.08 ± 0.08 ^b^	2.28 ± 0.19 ^d^	*
Citric acid (g/L)	*	*	*	*	*	*	*	*	*	0.44 ± 0.10
Glucose (g/L)	22.6 ± 1.6 ^bc^	22.2 ± 1.4 ^bc^	24.1 ± 1.5 ^c^	21.0 ± 1.1 ^b^	20.9 ± 0.9 ^b^	21.2 ± 1.0 ^b^	29.1 ± 1.5 ^d^	30.0 ± 1.6 ^d^	31.1 ± 1.7 ^d^	17.0 ± 0.7 ^a^
Fructose (g/L)	15.4 ± 0.8 ^cd^	16.1 ± 0.7 ^d^	16.5 ± 1.0 ^d^	13.6 ± 1.0 ^bc^	12.6 ± 0.7 ^ab^	11.6 ± 0.8 ^a^	15.3 ± 1.0 ^cd^	14.6 ± 0.9 ^cd^	12.4 ± 1.1 ^ab^	11.2 ± 0.8 ^a^
Sucrose (g/L)	69.4 ± 3.4 ^e^	37.26 ± 3.1 ^d^	20.6 ± 1.6 ^b^	66.7 ± 2.8 ^e^	33.2 ± 2.7 ^cd^	17.3 ± 1.8 ^ab^	67.8 ± 2.4 ^e^	31.6 ± 2.3 ^c^	14.3 ± 1.1 ^a^	92.1 ± 3.2 ^f^
Potassium (g/L)	1.51 ± 0.10 ^a^	1.48 ± 0.12 ^a^	1.57 ± 0.14 ^ab^	1.78 ± 0.12 ^bc^	1.84 ± 0.14 ^bc^	2.01 ± 0.13 ^cd^	2.2 ± 0.13 ^de^	2.19 ± 0.15 ^de^	2.42 ± 0.12 ^e^	1.70 ± 0.14 ^abc^
Catechins (g/L)	0.23 ± 0.02 ^c^	0.24 ± 0.02 ^c^	0.23 ± 0.03 ^c^	0.19 ± 0.02 ^abc^	0.16 ± 0.03 ^ab^	0.17 ± 0.02 ^ab^	0.20 ± 0.02 ^bc^	0.20 ± 0.02 ^bc^	0.25 ± 0.02 ^c^	0.16 ± 0.02 ^a^
Total phenolics (g/L, gallic acid equivalent)	0.29 ± 0.04 ^bc^	0.27 ± 0.04 ^abc^	0.21 ± 0.03 ^a^	0.47 ± 0.05 ^d^	0.48 ± 0.06 ^d^	0.33 ± 0.04 ^c^	0.29 ± 0.03 ^bc^	0.32 ± 0.04 ^c^	0.22 ± 0.05 ^ab^	0.35 ± 0.05 ^c^
DPPH (g/L, gallic acid equivalent)	0.32 ± 0.03 ^b^	0.32 ± 35 ^b^	0.30 ± 0.04 ^ab^	0.36 ± 0.05 ^bc^	0.34 ± 0.04 ^bc^	0.34 ± 0.03 ^bc^	0.25 ± 0.04 ^a^	0.29 ± 0.04 ^ab^	0.25 ± 0.03 ^a^	0.41 ± 0.05 ^c^

*: below the detection limit (0.1 g/L). ^a–f^: For the same parameter, average values lacking a common letter (a,b…) significantly differ from each other.

**Table 4:** Volatile compounds (mean value expressed as ppm equivalent) detected through GC-MS-SPME in the different kombucha samples (P1, P2, and P3) during fermentation (T_0_, T_48_, and T_end_).

	ppm Equivalents									
Compounds	Samples									
		P1			P2			P3		Sugared Raw Tea
	T_0_	T_48_	T_end_	T_0_	T_48_	T_end_	T_0_	T_48_	T_end_	
Pentanal	-	-	-	-	-	-	-	-	-	0.07
Acetaldehyde	0.02	0.06	0.05	0.14	0.08	0.10	0.95	0.88	1.32	-
Hexanal	-	-	-	-	-	-	-	-	-	0.45
Nonanal	0.06	0.11	0.12	0.03	0.05	-	0.11	0.08	0.15	0.18
Decanal	-	-	-	-	-	-	-	-	-	0.07
2,4 Heptadienal (E,E)	-	-	-	-	-	-	-	-	-	0.33
Furfural	0.29	0.40	0.35	0.11	-	-	-	-	-	0.36
Benzaldehyde	-	-	-	-	-	-	0.10	-	-	0.09
Phenylacetaldehyde	-	-	-	-	-	-	0.16	0.14	0.05	-
Total aldehydes	0.37	0.58	0.52	0.28	0.13	0.10	1.32	1.10	1.52	1.54
Acetic acid	37.62	31.19	28.67	37.79	34.31	44.50	31.20	49.96	56.22	-
2-Methyl-propionic acid	-	0.17	-	-	0.28	-	-	-	-	-
Butanoic acid	0.19	0.26	-	-	-	-	-	-	-	-
Isopentanoic acid	1.08	1.09	0.96	0.84	0.91	1.22	0.68	0.90	0.62	-
Hexanoic acid	0.94	1.16	1.15	0.92	0.95	0.18	0.66	0.60	0.44	-
Propanoic acid, 2-methyl-	0.17	0.25	0.21	-	-	-	-	-	-	-
Caprylic acid	4.95	6.35	5.99	2.18	3.20	0.07	1.02	1.73	2.62	-
Pelargonic acid	0.23	0.41	0.30	0.07	0.10	0.06	0.12	0.09	0.03	-
Decanoic acid	1.55	2.36	1.81	0.03	0.56	0.08	0.24	0.40	0.72	-
Total acids	46.72	43.24	39.09	41.83	40.31	46.10	33.91	53.67	60.64	-
Methyl isobutyl ketone	0.32	0.37	0.29	0.34	0.30	0.27	0.39	0.32	0.58	0.49
3-Hexanone, 5-methyl-	-	-	-	-	-	-	0.09	-	-	0.09
2-Hexanone, 4-methyl-	0.13	0.14	0.13	0.11	0.11	0.02	0.16	0.13	0.25	0.30
4 Heptanone 2,6 dimethyl	-	-	-	-	-	-	-	-	-	1.24
Diisobutyl ketone	0.89	1.26	0.89	0.88	-	-	1.50	0.92	0.07	-
2-Heptanone	-	0.01	0.05	-	-	-	0.07	-	-	-
2-Heptanone, 4,6-dimethyl-	0.02	0.05	-	-	-	-	0.05	-	-	0.06
3-Hydroxy-2-butanone	-	-	-	-	-	-	0.05	0.10	-	-
Hydroxyacetone	-	0.08	0.05	-	-	-	0.02	0.01	0.17	-
3,5-Octadien-2-one	0.02	0.03	-	0.04	-	-	0.78	-	-	0.20
1,3-Cyclopentanedione	0.05	0.05	-	-	-	-	-	-	-	0.05
Total ketones	1.43	1.99	1.40	1.37	0.41	0.28	3.11	1.47	1.07	2.43
Ethanol	25.42	21.40	31.77	43.57	51.84	72.27	17.74	37.41	42.30	-
Isoamyl alcohol	0.65	0.71	1.04	1.23	1.76	1.63	0.53	0.88	0.61	-
2 Penten 1 ol (Z)	-	-	-	-	-	-	-	-	-	0.27
1-Hexanol	-	-	-	0.08	-	-	-	-	-	0.29
2-Hexanol	0.06	0.08	0.08	0.03	0.06	-	0.06	-	-	0.30
2-Ethylhexanol	0.21	0.20	0.17	-	-	-	1.23	0.07	0.04	0.11
1,6-Octadien-3-ol, 3,7-dimethyl-	0.41	0.65	0.66	0.29	0.28	0.12	0.09	0.54	0.35	0.66
Alpha.-Terpineol	0.55	0.65	0.81	0.38	0.31	-	0.68	0.63	0.15	-
Phenylethyl alcohol	3.50	4.79	5.89	5.40	4.57	3.97	7.23	7.90	4.55	-
4-Ethylguaiacol	0.31	0.34	0.41	0.11	0.86	0.83	-	-	-	-
4-Ethylphenol	0.20	0.27	0.32	0.67	0.88	0.84	-	-	-	-
2,4-Di-tert-butylphenol	0.36	0.43	0.26	0.27	0.12	0.11	0.54	0.47	0.14	0.26
Farnesol	0.58	0.66	0.38	0.31	-	-	-	-	-	-
Total alcohols	32.25	30.18	41.80	52.34	60.69	79.77	28.11	47.89	48.14	1.89
Ethyl acetate	4.51	4.65	6.51	4.98	4.18	3.68	4.77	9.01	3.53	-
Ethyl hexanoate	-	-	-	-	0.11	-	-	-	-	-
Ethyl octanoate	0.17	0.28	0.48	2.90	2.83	1.30	0.10	0.06	0.07	-
Ethyl decanoate	0.10	0.16	0.29	1.39	0.71	0.53	-	-	-	-
Ethyl 9-hexadecenoate	-	0.05	0.09	0.52	0.77	0.29	-	-	-	-
2-Phenylethyl acetate	0.24	0.29	0.39	0.47	0.43	0.33	0.30	0.38	0.20	-
Butyl butanoate	0.41	0.52	0.46	0.40	0.27	0.28	0.17	0.16	0.08	0.11
Total esters	5.43	5.94	8.23	10.66	9.31	6.40	5.34	9.60	3.88	0.11
Other molecules	0.87	1.14	0.94	0.77	0.72	0.51	1.09	0.88	0.85	0.16
Total molecules	87.1	83.2	92.0	107.3	111.6	133.2	72.9	114.6	116.2	6.1

The coefficients of variability, expressed as the percentage ratios between the standard deviations and the mean values, ranged between 2% and 5%. “-”: not detected.

## Data Availability

The original contributions presented in the study are included in the article, further inquiries can be directed to the corresponding author.
